# *In vivo* autophagy and biogenesis of autophagosomes within male haploid cells during spermiogenesis

**DOI:** 10.18632/oncotarget.18221

**Published:** 2017-05-26

**Authors:** Ping Yang, Nisar Ahmed, Lingling Wang, Hong Chen, Yasir Waqas, Tengfei Liu, Abdul Haseeb, Nasrullah Bangulzai, Yufei Huang, Qiusheng Chen

**Affiliations:** ^1^ Laboratory of Animal Cell Biology and Embryology, College of Veterinary Medicine, Nanjing Agricultural University, Nanjing 210095, China; ^2^ The Postdoctoral Research Station in Animal Science, College of Animal Science & Technology, Nanjing Agricultural University, Nanjing 210095, China; ^3^ Department of Veterinary Anatomy & Histology, Faculty of Veterinary and Animal Sciences, LUAWMS, Uthal 90150, Pakistan

**Keywords:** ATG7, LC3, male haploid cells, spermiogenesis, chrysanthemum flower center

## Abstract

Autophagy is a unique catabolic pathway that is linked to several physiological processes. However, its role in the process of spermiogenesis is largely unknown. The aim of the current study was to determine the *in vivo* role of autophagy and the origin of autophagosome membrane biogenesis within male haploid cells. Our immunohistochemistry results demonstrated that LC3 and ATG7 localization were increased dramatically in round to elongated spermatids (haploid cells) towards the lumen of seminiferous tubules, however, poorly expressed in the early stages of germ cells near the basal membrane. Moreover, transmission electron microscopy revealed that the numbers of lysosomes and autophagosomes increased in the elongated spermatids as spermiogenesis progressed. However, no evidence was found for the presence of autophagosomes in the Sertoli cells, spermatogonia or early primary spermatocytes (diploid cells). Furthermore, TEM showed that many endoplasmic reticula were transformed into a “chrysanthemum flower center,” from which a double-layered isolation membrane appeared to develop into an autophagosome. This study provides novel evidence about the formation of autophagosomes through the chrysanthemum flower center from the endoplasmic reticulum, and suggests that autophagy may have an important role in the removal of extra cytoplasm within male haploid cells during spermiogenesis.

## INTRODUCTION

Spermiogenesis is a highly complex morphological process that lasts from the end of the meiosis to the release of mature sperm within the seminiferous tubule of the testis. During the process of spermiogenesis, the spermatids undergo a structural reorganization involving development of the acrosome, condensation of the nuclear material, rearrangement of the mitochondria and removal of unnecessary cytoplasm [1, 2]. All of the above events are interconnected by different cellular processes, and failure of any of these processes during the differentiation of spermatids can cause various abnormalities within the morphology of spermatozoa [3, 4]. Although these morphological changes during spermiogenesis have been well documented, the cellular mechanisms of unnecessary cytoplasm shedding and acrosomal changes are still unknown [5, 6]. Thus, we hypothesized that autophagy may be involved in spermiogenesis within male haploid germ cells.

Autophagy is an intercellular pathway that delivers cytoplasmic components or organelles to lytic compartments such as lysosomes (in mammals) or vacuoles (in yeast) for breakdown and recycling [7, 8]. First, a double-membraned cupped structure known as phagophore develops; it engulfs some portion of cytoplasm and then closes to form a double membrane-bound vesicle called the autophagosome. This process of autophagosome development consists of three major steps: initiation of membrane biogenesis, elongation of the isolation membrane, and closure of the membrane [9]. Autophagy is generally induced by starvation, but recent findings have linked it to numerous physiological and pathological conditions, such as normal development, placental detachment during birth in mammals, cancer, programmed cell death, neurodegenerative disorders, diabetes and infections [10–13].

The current biology of autophagy was revolutionized following the identification of so-called Atg (autophagy) genes within yeast, most of which are conserved in mammals [14, 15]. ATG7 is one particular member of the ATG protein family that acts as an E1-like activating enzyme that facilitates both LC3 and Atg12 [16]. LC3 is a microtubule-associated light protein chain 3, upon induction of autophagy LC3-I is conjugated to phosphatidylethanolamine to form LC3-II, which is tightly bound to the membrane of the autophagosome. Microtubule-associated protein 1 light chain 3-II is a marker of autophagosomes [17].

The origins of autophagosome membrane biogenesis may involve various sources [18, 19], including ER exit sites (ERES) [20, 21], the ER–Golgi intermediate compartment (ERGIC) [22], the Golgi [23, 24], the recycling endosome [19, 25, 26] and the plasma membrane [9]. Despite significant progress in identifying molecules (Atg genes) responsible for autophagosome formation, the origin and exact source of the autophagosomal membrane are still unclear [9]. Thus, the objectives of the current study were to determine the *in vivo* role of autophagy and origin of autophagosome membrane biogenesis within male haploid germ cells during spermiogenesis in the rat testis.

## RESULTS

### LC3 and ATG7 are mainly localized in the male haploid cells

**Figure 1:**
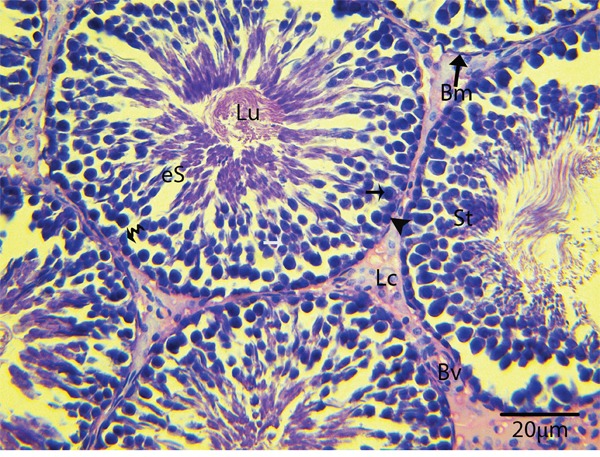
Light micrograph showing the histological structure of rat testis St: seminiferous tubule; Bm: basal membrane; Lu: lumen; eS: elongated spermatid; Lc: Leydig cell; Bv: blood vessels; (▲): spermatogonia; (black arrow): primary spermatocytes. H & E stain. Scale bar = 20μm.

**Figure 2:**
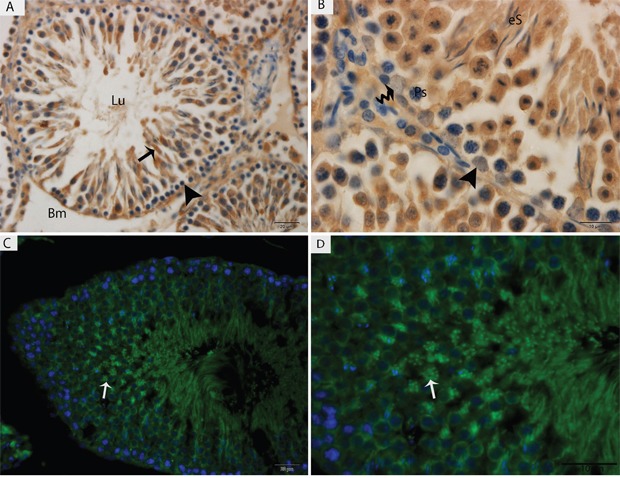
Light micrograph of LC3 localization in the rat testis **(A, B)** Immunohistochemistry, **(C, D)** immunofluorescence. Lu: lumen; Bm: basal membrane; Ps: primary spermatocytes; eS: elongated spermatid; (▲): spermatogonia. Scale bars = 20μm **(A, C)** and 10μm **(B, D).**

**Figure 3:**
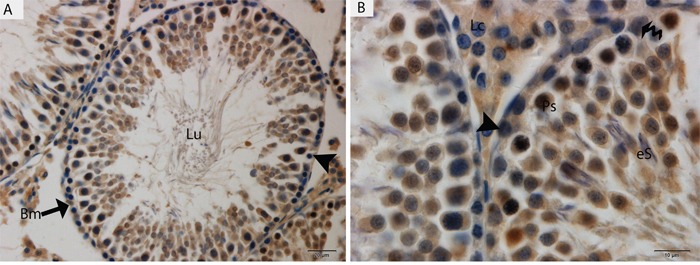
Light micrograph of ATG7 localization in the rat testis Lu: lumen; Bm: basal membrane; Ps: primary spermatocytes; eS: elongated spermatid; Lc: Leydig cell; (▲): spermatogonia. Scale bars = 20μm **(A)** and 10μm **(B).**

Light microscopy revealed that the seminiferous tubules (STs) of rat testes contained different stages of developing germ cells and Sertoli cells. Spermatogonia were found near the basal membrane and primary spermatocytes, including some early round spermatids that were located in the basal compartments of the STs. Elongated spermatids were observed near the lumens of STs. The interstitial space between STs contained numerous Leydig cells and blood vessels (Figure 1). IHC for LC3 within the seminiferous tubules showed no positive localization around the basal membranes within the spermatogonia or primary spermatocytes or on the Sertoli cells. While weak immunoreactivity was noted in the round spermatids and high expression was found on elongated spermatids, localization increased from the basal to the luminal compartments, corresponding with the progression of spermiogenesis (Figure 2A and 2B). Similarly, immunofluorescence for GPF-LC3 revealed a strong focal localization around spermatids and progressively increased in elongated spermatids towards the lumen, this focal localization (light green spots) indicates the autophagosomes (Figure 2C and 2D). Furthermore, ATG7 showed no localization on spermatogonia or Sertoli cells; weak positive expression was noted in the primary spermatocytes and in the round spermatids around their basal compartments. Immunoreactivity was highly increased within the elongated spermatids near their lumens, as observed for LC3 (Figure 3A and 3B).

### Lysosomes and autophagosomes appear within the male haploid germ cells

**Figure 4:**
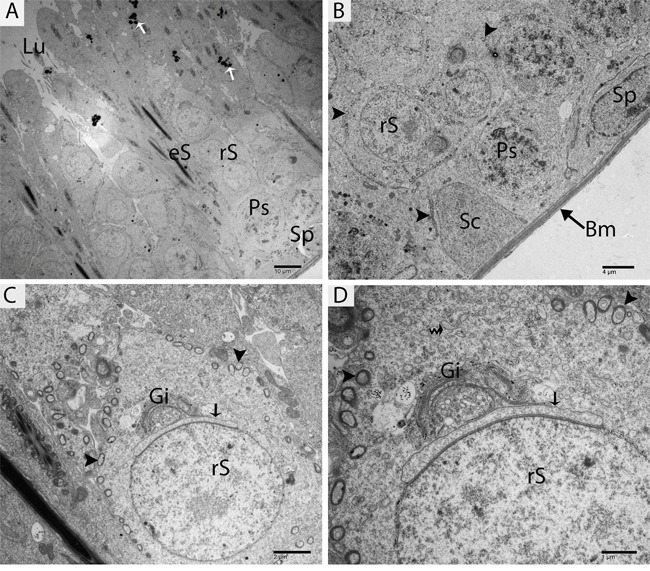
TEM micrograph of a germ cell at an early stage of development and Sertoli cells Sp: spermatogonia; Ps: primary spermatocytes; rS: round spermatid; eS: elongated spermatid; SC: Sertoli cell; Bm: basal membrane; Lu: lumen; Gi: Golgi apparatus; (▲): mitochondrion; (arrow): acrosomal vesicle; (curved arrow): endoplasmic reticulum; (white arrow): lysosome. Scale bars = 10μm **(A)**, 4μm **(B)**, 2μm **(C)** and 1μm **(D).**

**Figure 5:**
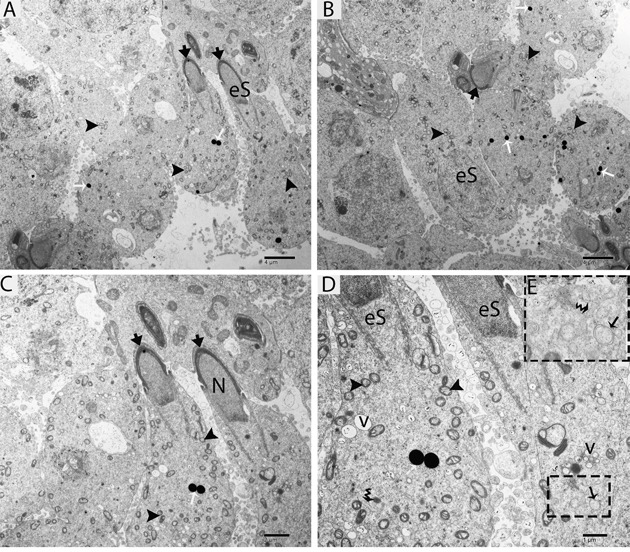
TEM micrograph of an elongated spermatid at an early stage showing lysosomes and autophagosomes eS: elongated spermatid; (arrowhead): acrosome; N: nucleus; v: vesicle; (▲): mitochondrion; (black arrow): autophagosome; (white arrow): lysosome; (curved arrow): endoplasmic reticulum. Scale bars =4μm **(A, B)**, 2μm **(C)** and 1μm **(D).**

**Figure 6:**
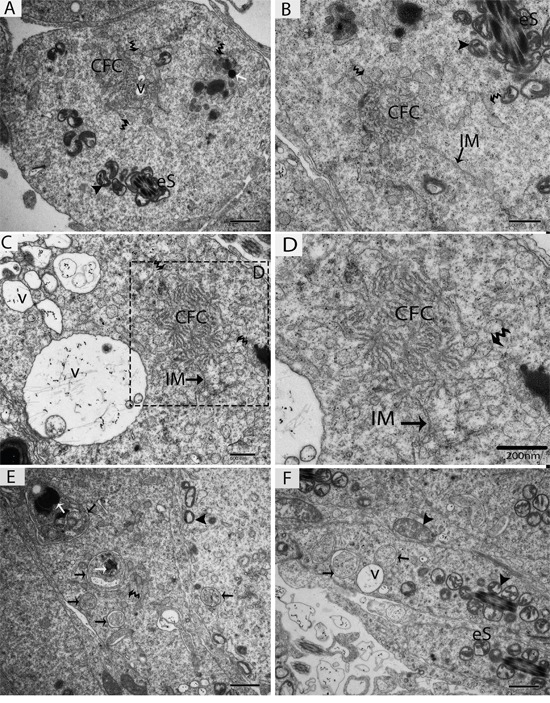
TEM micrograph of a late stage of spermiogenesis showing autophagosome biogenesis eS: elongated spermatid; CFC: chrysanthemum flower center; IM: isolation membranes; v: vesicle; (▲): mitochondrion; (black arrow): autophagosome; (white arrow): lysosome; (curved arrow): endoplasmic reticulum. Scale bars = 1μm **(A, E, F)** and 600nm **(B, C,)** and 200nm **(D).**

**Figure 7:**
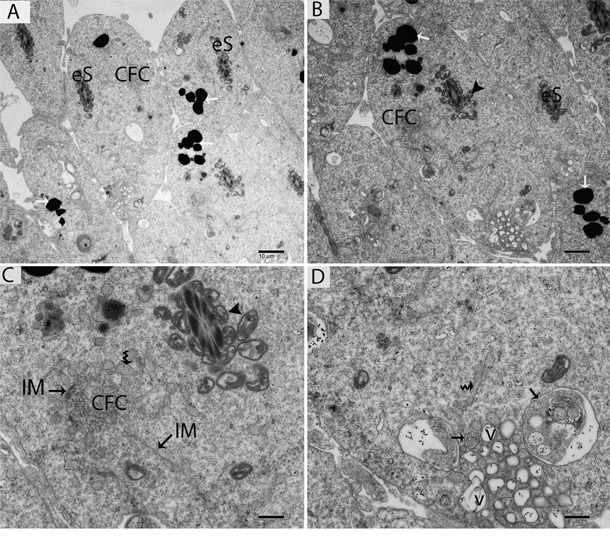
TEM micrograph of an elongated spermatid showing large numbers of lysosomes and autophagosomes near the lumen of a seminiferous tubule eS: elongated spermatid; CFC: chrysanthemum flower center; v: vesicle; (▲): mitochondrion; (black arrow): autophagosome; (white arrow): lysosome; (curved arrow): endoplasmic reticulum; IM: isolation membranes. Scale bars = 10μm **(A)**, 5μm **(B)**, 2μm **(C)** and 1μm **(D).**

**Figure 8:**
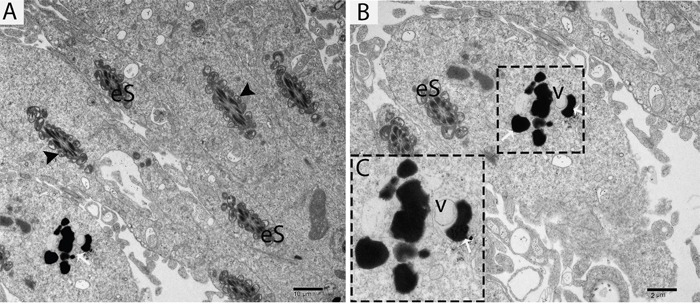
TEM micrograph of an elongated spermatid showing a vesicle attached to a lysosome eS: elongated spermatid; v: vesicle; (▲): mitochondrion; (white arrow): lysosome. Scale bars = 10μm **(A)**, 2μm **(B)** and 500nm **(C).**

TEM findings revealed that the STs of rat testes consist of Sertoli cells, spermatogonia, primary spermatocytes and round spermatids within the basal compartment, while elongated spermatids and several lysosomes are located near the luminal compartment (Figure 4A). Spermatogonia and Sertoli cells were found to rest directly on the basal membranes, and their cytoplasm contained extremely long mitochondria. Mitochondria within the round spermatids were vacuolated and either round or oval; moreover, these mitochondria lined up within the cytoplasm near the periphery of cell membrane. Acrosomal vesicles were attached to the nuclei of round spermatids, indicating the beginning of acrosome formation; meanwhile, Golgi apparatuses and endoplasmic reticula were also observed (Figure 4B, 4C and 4D). During further formation of the acrosome and progression of nuclear elongation, mitochondria were found to be scattered within the cytoplasm and moving toward the mid-piece of the elongated spermatids. At this stage, autophagosomes and some lysosomes appeared within the cytoplasm of elongated spermatids (Figure 5A, 5B, 5C and 5D). The elongated spermatids, which were located near the lumens of the STs, contained large size lysosomes. Meanwhile, the number of autophagosomes also increased within the cytoplasm of these elongated spermatids as spermiogenesis progresses, corresponding to the latter stages of spermiogenesis. At this stage, mitochondria lined up around the mid-piece and appeared dense (Figures 6, 7, 8).

### Origin of autophagosome membrane biogenesis

Further TEM analysis revealed that autophagosome membrane formation originated in the endoplasmic reticulum (ER) of differentiating spermatids. From the ER, a structure developed whose name we coined as the “chrysanthemum flower center” (CFC) because it resembled a chrysanthemum flower. Numerous branches of the ER were distributed around the CFC, and the ER ends attached to the CFC through short narrow tubules that formed many daisy petal-like structures. Meanwhile, several vesicles were found between and around the CFC (Figures 6B, 6C, 6D, 6E, 7B and 7C). Denser double-layer isolation membranes (IM) appeared in the CFC, and these membranes elongate and enwrap cytoplasm along with different structures to develop into autophagosomes as previously reported by our research group in the turtle [5]. Autophagosomes were attached to vesicles and lysosome; lysosomes and vesicles were also found inside the autophagosome along with other structures (Figures 6E, 6F and 7D). The lysosome was found closely attached to the vesicle membranes located near the lumen of ST in elongated spermatids (Figure 8A and 8B).

## DISCUSSION

During spermiogenesis, efficient removal of cytoplasm is thought to be important for the generation of motile and functional spermatozoa, but this mechanism remains poorly understood [27]. Previously proposed mechanisms suggest that the elimination of residual bodies and cytoplasm are mediated by the Sertoli cells via engulfment [28, 29]. However, the role of the germ cells in this process is largely understudied [30]. In the current work, we found that *in vivo* autophagy developed within haploid germ cells as elongation progressed. Our immunohistochemistry for LC3 and ATG7 showed expression increased dramatically in elongated spermatids as spermiogenesis progresses from basal toward the luminal compartment of ST. This expression pattern suggests that autophagy might be involved in some process in elongated spermatids. Shang and his colleagues [30] reported similar patterns of expression of LC3A/B and ATG7 in germ cell-specific *atg7* knockout mice, which exhibit multiple abnormalities in their spermatozoa. In addition, we also found some positive signals of ATG7 within the primary spermatocytes. Recently, reported that the depletion of ATG7 in germ cells did not affect the early stages of development of germ cells in the Atg7-knockout mice [6]. The cytoskeleton-based structures, such as F-actin-containing cytoplasmic manchettes, are also disrupted after the ablation of ATG7, consequently impairing acrosome formation and preventing complete cytoplasm removal. ATG7 is an E1-like activating enzyme that plays an essential role in the 2 ubiquitin-like conjugation system of autophagy. It is essential for ATG12 conjugation and for LC3 association with the membrane [12].

Further, our transmission electron microscopy showed that lysosomes and autophagosomes appeared within elongated spermatids during acrosome formation and that their number increased as elongation progressed toward the lumens of seminiferous tubules. Similar to our immunohistochemistry (LC3 and ATG7) findings, TEM showed no consistent evidence of autophagy within the Sertoli cells or early germ cell stages (spermatogonia, primary spermatocytes and early round spermatids). These results suggest that these autophagosomes and lysosomes might have role in removal of extra cytoplasm from the spermatids during spermiogenesis. Moreover, autolysosomes are essential for acrosome development, while mere lysosomes are not. Acrosome biogenesis is highly disrupted during early stages of spermiogenesis in spermatids, as the proacrosomal vesicles are unable to fuse into a single acrosomal vesicle during the Golgi phase secondary to ATG7 depletion [6]. Together, these findings collectively suggest that the autophagy is an essential for the process of spermiogenesis.

Autophagy depends on the formation of double-membraned autophagosomes. Unlike other organelles that appear to stably exist in the cell, autophagosomes only appear when required [31]. It is difficult to confirm the sources of autophagosomal membranes because autophagosome formation occurs very rapidly once initiated. It is estimated that autophagosome formation takes only a few minutes in yeast and mammals [11, 24, 32]. How and where this dynamic autophagosome formation occurs is still unknown [8]. The TEM findings of the current study revealed a novel structure that we named the “chrysanthemum flower center” (CFC). The CFC developed from the endoplasmic reticulum (ER), and then isolation membranes (phagophores) arose from the CFC. Phagophore elongate and envelop the cytoplasm to develop autophagosomes. We recently reported the more clear evidence of chrysanthemum flower center in the spermatids of Chinese soft-shelled turtle, because it is better model to study different events of spermiogenesis then the rat. The CFC was more developed in spermatids with compact nuclei than in spermatids with granular nuclei in the turtle [5].

The current work showed that the ER might be a basic source of autophagosome membrane biogenesis. A strong relationship between ER and autophagosome formation sites have been observed in mammalian cell culture [8]. Additionally, 70% of isolation membranes (IM) were associated with the ER (named ER–IM complexes) in Atg4B (Atg4BC74A) mutant cells [33]. In higher eukaryotes, phagophores nucleate at an intricate membranous structure called the omegasome, which was originally identified as being associated with the ER [34]. Our findings are consistent with above studies, while additionally revealing for the first time that the CFC is a novel structure that develops from the ER and that the IM develops from the CFC. Moreover, recent studies suggest that endoplasmic reticulum exit sites (ERES), specialized ER regions where proteins are sorted into the secretory system, are key players in the formation of autophagosomes. Therefore, ERES are a new element that should be integrated into descriptions of autophagosome biogenesis [35]. In yeast, the membrane of the phagophore originates from a structure near the vacuole called the pre-autophagosomal structure (PAS) [36]. We also found several vesicles at the different stages of autophagosomes biogenesis.

In conclusion, the numbers of lysosomes, CFCs, phagophores and autophagosomes increase within male haploid cells as spermiogenesis progresses. Additionally, the endoplasmic reticulum might be a source of autophagosome membrane before phagophore formation via a newly described structure that we refer to as the “chrysanthemum flower center” (CFC). These findings may initiate a new area of focus for understanding the origin of the autophagosome membrane.

## MATERIALS AND METHODS

### Animals

Six three-month-old male albino rats were purchased from Nanjing, Jiangsu Province, China for this study. Rats were initially weighted (170-200 gm) and were reared for 10 days under a pathogen-free environment. Then, all rats were rendered comatose using intraperitoneally administered sodium pentobarbital (20 mg/animal) and were then sacrificed by cervical dislocation. The testes were collected immediately and fixed to performed different techniques (details below). Sample preparation was conducted according to accepted international standards and was approved by the Ethics Committee for Animal Care and Use by the Science and Technology Agency of Jiangsu Province (SYXK (SU) 2010-0009).

### Light microscopy

The testes were fixed in 10% neutral buffered formalin overnight, and then paraffin blocks were prepared. Sectioning was performed at 6μm. The sections were stained with hematoxylin and eosin procedures (Harry's hematoxylin for 2 min and 1% eosin for 30 sec) for light microscopic analysis using an Olympus microscope (BX53), camera (Olympus DP73, Japan).

### Immunohistochemistry (IHC)

Immunohistochemical staining for LC3 and ATG7 was performed according to the manufacturer's recommendations and as suggested in previous studies [37, 38]. The sections were processed using a standard immunohistochemistry protocol as previously described [39]. Briefly, after deparaffinization, blocking endogenous peroxidase, microwave antigen retrieval, and BSA (bovine serum albumin) blocking, a primary rabbit anti-LC3B polyclonal antibody (ab48394, Abcam, Cambridge, UK) or mouse anti-ATG7 monoclonal antibody (SAB4200304, Sigma Aldrich, St. Louis, USA) was applied for 1 h at room temperature. After washing with PBS, the slides were incubated for 30 min with biotinylated goat anti-rabbit antibody. Peroxidase was visualized with DAB, and the sections were counterstained with hematoxylin. Sections incubated in PBS alone served as negative controls.

### Fluorescence microscopy

After deparaffinization, serum blocking and antigen retrieval, the tissue sections of testis were incubated with the primary antibody (LC3) at 4°C overnight. Next, the slides were washed in PBS and corresponding secondary antibodies were added to sections for 2 h at room temperature. After washing again with PBS, then DAPI stain to mark the nuclei. Images were taken immediately using an Olympus microscope (BX53), camera (Olympus DP73), Japan.

### Transmission electron microscopy (TEM)

Samples were cut into small pieces and then immersed in 2.5% glutaraldehyde in PBS (4°C, pH 7.4, 0.1 M) overnight. Tissues were rinsed in PBS and then post-fixed for 60 min at room temperature in the same way by using buffered 1% osmium tetroxide (Polysciences Inc. Warrington, PA, USA) and then washed in the buffer. The samples were then dehydrated in ascending concentrations of ethyl alcohol, infiltrated with a propylene oxide-araldite mixture and then embedded in araldite. The blocks were then sectioned using an ultramicrotome (Reichert Jung, Wien, Austria), and the ultrathin sections (50 nm) were mounted on copper-coated grids. The pieces were stained with 1% uranyl acetate and Reynold's lead citrate for 20 min. Finally, the samples were examined and photographed using a high-resolution digital camera (16 mega pixel) connected to a TEM (Hitachi H-7650, Japan).
